# European society of intensive care medicine study of therapeutic hypothermia (32-35°C) for intracranial pressure reduction after traumatic brain injury (the Eurotherm3235Trial)

**DOI:** 10.1186/1745-6215-12-8

**Published:** 2011-01-12

**Authors:** Peter JD Andrews, Helen Louise Sinclair, Claire G Battison, Kees H Polderman, Giuseppe Citerio, Luciana Mascia, Bridget A Harris, Gordon D Murray, Nino Stocchetti, David K Menon, Haleema Shakur, Daniel De Backer

**Affiliations:** 1 Department of Anaesthesia, Critical Care and Pain Management, University of Edinburgh, UK; 2 Critical Care Medicine, 3550 Terrace Street, Pittsburgh, Pennsylvania PA 15261, USA; 3 Neurorianimazione, Dipartimento di Anestesia e Rianimazione, Nuovo Ospedale San Gerardo, Via Pergolesi 33, 20052 Monza (MI), Italy; 4 Dipartimento di Anestesiologia e Rianimazione, Università di Torino, Ospedale S. Giovanni Battista, Torino, Italy; 5 Public Health Sciences section, Division of Community Health Sciences, The University of Edinburgh, Medical School, Teviot Place, Edinburgh, EH8 9AG, UK; 6 Terapia Intensiva Neuroscienze, Ospedale Maggiore, Policlinico IRCCS, Via S Sforza, 3520 122 Milan, Italy; 7 Department of Anaesthesia, Division of Anaesthesia, University of Cambridge, UK; 8 Clinical Trials Unit, London School of Hygiene and Tropical Medicine, Keppel Street, London WC1E 7HT, UK; 9 Erasme University Hospital, Free University of Brussels, 808 Route de Lennick Brussels, B-1070, Belgium

## Abstract

**Background:**

Traumatic brain injury is a major cause of death and severe disability worldwide with 1,000,000 hospital admissions per annum throughout the European Union.

Therapeutic hypothermia to reduce intracranial hypertension may improve patient outcome but key issues are length of hypothermia treatment and speed of re-warming. A recent meta-analysis showed improved outcome when hypothermia was continued for between 48 hours and 5 days and patients were re-warmed slowly (1°C/4 hours). Previous experience with cooling also appears to be important if complications, which may outweigh the benefits of hypothermia, are to be avoided.

**Methods/design:**

This is a pragmatic, multi-centre randomised controlled trial examining the effects of hypothermia 32-35°C, titrated to reduce intracranial pressure <20 mmHg, on morbidity and mortality 6 months after traumatic brain injury. The study aims to recruit 1800 patients over 41 months. Enrolment started in April 2010.

Participants are randomised to either standard care or standard care with titrated therapeutic hypothermia. Hypothermia is initiated with 20-30 ml/kg of intravenous, refrigerated 0.9% saline and maintained using each centre's usual cooling technique. There is a guideline for detection and treatment of shivering in the intervention group. Hypothermia is maintained for at least 48 hours in the treatment group and continued for as long as is necessary to maintain intracranial pressure <20 mmHg. Intracranial hypertension is defined as an intracranial pressure >20 mmHg in accordance with the Brain Trauma Foundation Guidelines, 2007.

**Discussion:**

The Eurotherm3235Trial is the most important clinical trial in critical care ever conceived by European intensive care medicine, because it was launched and funded by the European Society of Intensive Care Medicine and will be the largest non-commercial randomised controlled trial due to the substantial number of centres required to deliver the target number of patients. It represents a new and fundamental step for intensive care medicine in Europe. Recruitment will continue until January 2013 and interested clinicians from intensive care units worldwide can still join this important collaboration by contacting the Trial Coordinating Team via the trial website http://www.eurotherm3235trial.eu.

**Trial registration:**

Current Controlled Trials ISRCTN34555414

## Background

Traumatic brain injury (TBI) is a major cause of death and severe disability throughout the world. TBI leads to 1,000,000 hospital admissions per annum throughout the European Union. It causes the majority of the 50,000 deaths from road traffic accidents and leaves 10,000 patients severely handicapped: three quarters of these victims are young people [1]. Additionally, TBI causes 290 000 hospital admissions, 51 000 deaths and leaves 80 000 patients with permanent neurological disabilities in the United States annually [2]. The consequence of this is both a devastating emotional and physical impact and an enormous financial burden [3].

Therapeutic hypothermia has been shown to improve outcome after cardiac arrest [3], consequently the European Resuscitation Council and American Heart Association guidelines [4,5] recommend the use of hypothermia in these patients. Hypothermia is also thought to improve neurological outcome after neonatal birth asphyxia [6]. Cardiac arrest and neonatal asphyxia patient populations present to health care services rapidly and without posing a diagnostic dilemma, therefore therapeutic systemic hypothermia may be implemented relatively quickly. As a result of this, hypothermia in these two populations is similar to laboratory models where systemic therapeutic hypothermia is commenced very soon after the injury and has shown so much promise [7].

The need for resuscitation and Computerised Tomography (CT) imaging to confirm the diagnosis in patients with TBI, are factors which delay intervention with temperature reduction strategies. Treatments in TBI have traditionally focussed on restoring and maintaining adequate brain perfusion, surgically evacuating large haematomas where necessary, and preventing or promptly treating oedema [3]. Brain swelling can be monitored by measuring intracranial pressure (ICP), and in most centres ICP is used to guide treatments and to monitor their success. The use of hypothermia in TBI should be regarded in this context.

### Pathophysiology

Ischaemia has a key role in all forms of brain injury and preventing ischaemic (or secondary) injury is at the core of all neuroprotective strategies [3]. A complex cascade of processes ensues at the cellular level after a period of ischaemia beginning from minutes to hours after injury and continuing for up to 72 hours or longer. Thus, there may be a window of opportunity of several hours, or even days, during which injury can be mitigated by treatments such as hypothermia [3].

### Review of Clinical Evidence

In total, 29 clinical studies have been performed to assess the effects of hypothermia in TBI. Twenty-seven of these were performed in adult patients, 18 of which included control groups. Data from one pilot study were subsequently included in a larger study, therefore leaving 17 studies. As outlined above, study protocols have differed considerably, and not all studies were (properly) randomised [3]. A total of 131 patients were enrolled into two studies undertaken in patients with normal ICP. Only one of these studies reported outcome data (at 3 months) and the results showed no significant difference between groups (good outcome in 21/45 (hypothermia) versus 27/46 patients (controls), p = 0.251) [8].

**Figure 1:**
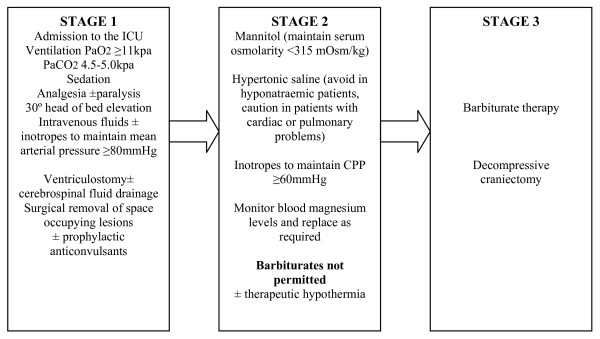
**Stages of therapeutic management of raised intracranial pressure after traumatic brain injury **[37,43].

Eighteen studies, with outcome data available for 2096 patients, used hypothermia in patients with high ICP that was refractory to "conventional" treatments (usually sedation/analgesia, muscle relaxants, osmotic therapy, and sometimes barbiturates) [9-26]. The results are summarised in Figure 1. All observed decreases in ICP during cooling. Thirteen of these studies reported significant improvements in outcome associated with hypothermia [10,12-14,16,17,19-25]. All of these were performed in specialised neurotrauma centres, with experience in applying hypothermia and managing its side effects. Ten were single centre studies [10,12,14,16,17,20,22-25], three (all performed in China) [14,20,22] were multi-centre. Four additional studies [11,15,18,21] observed a trend to improved outcome, but these differences were not statistically significant.

Interpretation of these results is complicated by the fact that these studies have enrolled different categories of patients, with different types of injuries, and have used widely diverging treatment protocols [27]. Most have used elevated ICP as an inclusion criterion although some have used CT-scan criteria. The duration of cooling varied from 24 hours to more than five days and re-warming rates have also varied. Some studies have used ICP to guide depth and duration of treatment although responses to rebound intracranial hypertension have differed [3]. Use of co-interventions such as osmotic therapy, sedation, analgesia, paralysis, targets for mean arterial pressure and cerebral perfusion pressure (CPP) have also varied considerably [3]. All of these factors can affect outcome after TBI in general, and the potential efficacy of cooling in particular. Thus interpreting, comparing and aggregating the results of these studies presents a number of complex challenges.

In contrast, one of the two largest multi-centre randomised controlled trials (RCTs) failed to show that therapeutic hypothermia improved outcome at 6 months after TBI (Relative Risk (RR) of a poor outcome 1; 95% CI 0.8-1.2; p = 0.99) [9]. Significantly more of the patients admitted to hospital with hypothermia who were randomised to normothermia, and consequently re-warmed, had a poor outcome (78% n = 31). Compared to patients admitted with hypothermia and treated with hypothermia (61% n = 38) (p = 0.09).

On subsequent analysis, it became clear that although this study was methodologically well designed, there was marked inter-centre variance in the treatment effect of hypothermia, age of participants, severity of illness scoring between groups, management of intracranial hypertension and haemodynamic and fluid management [28]. Therapeutic hypothermia in the hypothermia group was started relatively late with a slow speed of cooling (average time to target temperature >8 hours) in all centres.

Hypotension (lasting >2 hours) and hypovolaemia occurred three times more frequently in the hypothermia group. Bradycardia associated with hypotension also occurred four times more frequently in this group, electrolyte disorders and hyperglycaemia were also found more frequently in the hypothermia group [9]. All of these complications are known side effects of hypothermia. Most are easily preventable with good intensive care and should not be regarded as inevitable consequences of hypothermia treatment. Since even very brief episodes of hypotension or hypovolaemia can adversely affect outcome in TBI, these and other issues may have significantly affected the results of this trial [29-31]. One possible problem was that some of the participating centres had little or no previous experience in using hypothermia. Large centres, familiar with cooling, showed apparently favourable neurological outcomes whereas smaller centres showed poor outcomes.

### Induction of Hypothermia

The most widely accepted use of hypothermia in adults is after cardiac arrest. Two RCTs in this patient group have shown significant neurological improvements in patients treated with hypothermia many hours after injury, whose initial cardiac rhythm was ventricular fibrillation or ventricular tachycardia [32,33]. Subsequent data from a large study of patients after myocardial infarction suggest that infarct size was reduced in patients who were cooled to <35°C before coronary intervention [34]. This suggests that faster cooling rates may be beneficial to patient outcome.

Methods of cooling can be broadly divided into surface and core cooling techniques [35]. The study of patients after myocardial infarction used surface cooling devices alone and found that large numbers of patients did not reach target temperature quickly enough before the start of the coronary intervention [34]. Despite advancing technology in surface cooling devices and the introduction of endovascular catheters for core cooling, average periods of 2-3 hours are still required to reach temperatures of 32-34°C [35]. The currently available surface cooling devices are also relatively large and cumbersome. This, coupled with the need for staff with specialist knowledge of the management of therapeutic hypothermia, may prevent its use outside of an Intensive Care Unit (ICU) [35].

A recent study examined the feasibility, speed and complication rates of infusing refrigerated fluids intravenously to quickly induce hypothermia in patients with various neurological injuries [35]. Results showed that a 1500 ml infusion of 0.9% saline, administered over 30 minutes, in patients without cardiac shock, reduced core temperature from 36.9 ± 1.9°C to 34.6 ± 1.5°C at 30 minutes and to 32.9 ± 0.9°C at 60 minutes. Continuous monitoring of arterial blood pressure, heart rhythm, central venous pressure, arterial blood gasses and serum levels of electrolytes, platelets and white blood cells showed no significant adverse events [35].

When hypothermia develops, the body will immediately try to counteract the temperature drop to maintain homeostasis [36]. One of the key mechanisms of heat production is shivering which can lead to an increased oxygen consumption of 40%-100% which may be detrimental in this patient population. Sedation drugs are known to increase peripheral blood flow which, in turn, will increase the transfer of heat from the core to the peripheries, thus reducing core temperature [36]. Therefore shivering may be counteracted by the administration of sedatives, anaesthetic agents, opiates and/or muscle relaxants [36].

It should be noted however, that the capacity and effectiveness of the mechanisms of controlling body temperature decrease with age. Younger patients will therefore react earlier and with greater intensity than older patients. For this reason, induction of hypothermia in younger patients often requires high doses of sedation drugs to counteract the counter-regulatory mechanisms [36].

### Meta-analyses

Six meta-analyses have been published between the years 2000 and 2008 [37-42]. These included various numbers of trials, with varying quality of randomisation and blinding procedures. All found a trend to positive effects of hypothermia on neurological outcome, although statistical significance was reached in only two reviews: RR of improved neurological outcome 0.78 (95% CI 0.63-0.98) [37] and RR 0.68 (95% CI 0.52-0.89) [38].

The most recent meta-analysis [42] included eight trials which studied comparable patient groups at baseline. Hypothermia was shown to reduce mortality by 20% although this was not statistically significant (RR 0.80; 95% CI 0.59-1.09). Subgroup analysis showed that this effect was significantly greatest when hypothermia was maintained for >48 hours (RR 0.51; 95% CI 0.33-0.79). Hypothermia was also associated with a non-significant increase of 25% in neurological outcome when measured by the Glasgow Outcome Scale (GOS) at 6 months (RR 1.25; 95% CI 0.96-1.62). Despite not reaching statistical significance, results showed an increased likelihood of improved neurological outcome when cooling was maintained for >48 hours (RR 1.91; 95% CI 1.28-2.85). Another key finding of this meta-analysis was that hypothermia was only of significant benefit to those patients who had not received barbiturate therapy (RR 0.58 95% CI 0.40-0.85).

A criticism of these meta-analyses is that most failed to take account of important differences in patient groups (such as those with or without intracranial hypertension) and of differences in treatment protocols, except the use of hypothermia. Only one differentiated between studies that enrolled patients with normal ICP and those that enrolled patients with intracranial hypertension and found no neurological improvement associated with hypothermia [41]. Two assessed effects of treatment duration and speed of re-warming [37,38], concluding that cooling for >48 hours and re-warming rates of 24 hours, or 1°C/4 hours, were both key factors in reducing mortality (RR 0.70; 95% CI, 0.56-0.87) and improving neurological outcome (RR, 0.79; 95% CI 0.63-0.98) respectively.

### Rationale for Study

The evidence from previous research shows that treatment with therapeutic hypothermia to reduce intracranial hypertension may improve patient outcome after TBI. A recent meta-analysis has shown key relationships between the duration of hypothermia treatment and speed of re-warming with patient outcome [42]. Improved patient outcome was found when hypothermia was continued for between 48 hours and 5 days and patients were re-warmed slowly (1°C/4 hours). Experience with cooling also appears to be important if complications which may outweigh the benefits of hypothermia are to be avoided.

The Eurotherm3235Trial is examining the relationship between therapeutic hypothermia for ICP reduction after TBI and patient outcome. The trial is enrolling patients with TBI who have ICP >20 mmHg that is resistant to stage 1 therapy (Figure 1).

The Brain Trauma Foundation's recommended threshold for treatment of ICP is 20 mmHg [37,43]. Although early cooling after injury is considered to be beneficial, this is offset by failure to show benefit from hypothermia in the absence of raised ICP. Enrolment to the trial is therefore allowed for up to 72 hours following injury. This potential delay in cooling is compensated for, to an extent, by inducing hypothermia with 20-30 ml/kg of refrigerated 0.9% saline given intravenously over 20-30 minutes. No maximum duration of cooling is specified and hypothermia can continue until ICP is no longer dependent on temperature reduction to remain below 20 mmHg. Patients are then slowly re-warmed at a rate of 0.25°C per hour (1°C/4 hours).

The Extended Glasgow Outcome Scale (GOSE) is used to assess patient outcome at 6 months. Many patients with severe TBI are expected to have poor outcome. This outcome questionnaire is used as it is more sensitive to differences between poorer outcome categories after TBI than the 5-point Glasgow Outcome Score (GOS) [44,45].

## Study Objectives

### Hypothesis

Patients treated with therapeutic hypothermia (32-35°C) have reduced morbidity and mortality rates compared to those receiving standard care alone after TBI.

### Research Questions

Does therapeutic hypothermia (32-35°C) reduce morbidity and mortality rates at 6 months after TBI assessed by the GOSE questionnaire?

Does therapeutic hypothermia (32-35°C) reduce intracranial hypertension?

Is therapeutic hypothermia a costeffective treatment to improve outcome after TBI?

### Study Endpoints

#### Primary

• Outcome at 6 months using the GOSE questionnaire

#### Other

• 6 month mortality rate

• ICP control

• Incidence of pneumonia across both groups

• Length of stay in the ICU and hospital

• Modified Oxford Handicap Scale score at one month, discharge from the randomising hospital or death, whichever occurs first

• Correlation between the predicted outcome using the modified Oxford handicap scale at hospital discharge and the GOSE Score at 6 months post injury

• Health economics (dependent on additional external funding).

## Methods/design

**Figure 2:**
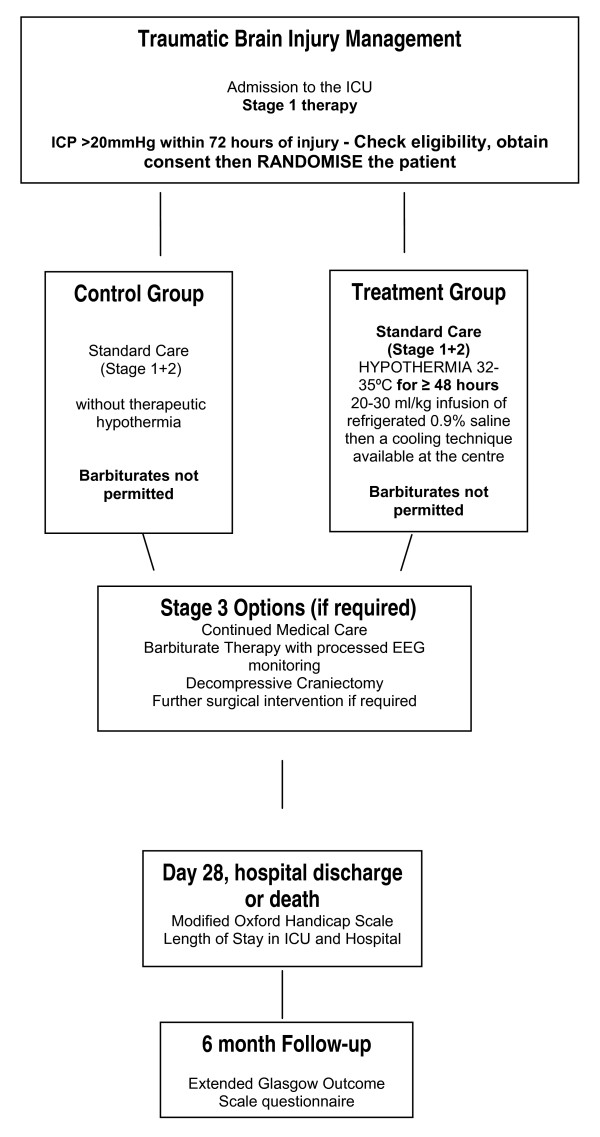
**Eurotherm3235Trial study flowchart**.

This is a pragmatic, multi-centre randomised controlled trial to examine the effects of hypothermia (32-35°C) on outcome after traumatic brain injury. The study is recruiting for 41 months. Participants are randomised to either the control or intervention group (Figure 2). Participants allocated to the control group receive standard care without therapeutic hypothermia. Participants randomised to the intervention group receive standard care with therapeutic hypothermia. Hypothermia is initiated with 20-30 ml/kg refrigerated 0.9% saline given intravenously and maintained using the cooling technique available at that centre. A flowchart has been designed for the induction and maintenance of therapeutic hypothermia in the intervention group. The depth of hypothermia (range: 32-35°C) is guided by ICP with a higher pressure level warranting a cooler target temperature. A guideline has been produced for the detection and treatment of shivering in the intervention group. This has been designed specifically for this trial drawing on:

• the hospital protocol of the Mission Hospital, Orange County California (permission given by Mary Kay Bader, Neuro Clinical Nurse Specialist, Mission Hospital, Orange County, Ca, USA)

• The hospital protocol of the University Medical Centre, Utrecht, The Netherlands (permission given by Dr Kees Polderman, UMC, Utrecht, The Netherlands)

• The Bedside Shivering Assessment Scale [46]

Therapeutic hypothermia of 32-35°C is maintained for at least 48 hours in the treatment group. Previous studies have shown that therapeutic hypothermia which lasts for at least 48 hours shows a trend to reduction in mortality and improved neurological function after TBI [42]. Hypothermia is continued for as long as is necessary to reduce and maintain ICP <20 mmHg. Intracranial hypertension is defined as an ICP >20 mmHg by the Brain Trauma Foundation Guidelines [37]. Together with therapeutic hypothermia, all patients in the intervention group continue to be treated with stage 1 and 2 therapies as required to reduce intracranial hypertension [37,38]. If raised ICP becomes resistant to these therapies and despite increasing the depth of hypothermia, care may be escalated to include stage 3 interventions. If this is required, therapeutic hypothermia treatment should be terminated for patients allocated to the treatment group and the patient re-warmed using the re-warming guideline. The reason for treatment escalation should be documented on the daily data collection form.

The primary endpoint of the Eurotherm3235Trial is outcome 6 months after TBI using the GOSE questionnaire. Participants are sent the GOSE questionnaire with a covering letter by post 6 months after randomisation by the coordinating centre.

### Study Population

#### Sample Size

A total of 1800 patients (900 per treatment group) will be enrolled. At least 70 hospitals specialising in the care of TBI patients will be initiated worldwide including centres in Belgium, Germany, Italy, Netherlands, Spain, Sweden and the United Kingdom.

#### Inclusion Criteria

1) Believed to be legal age for consent to take part in research to 65 years of age

2) Primary closed TBI

3) Raised ICP >20 mmHg for ≥5 minutes after first line treatments with no obvious reversible cause e.g. patient position, coughing, inadequate sedation

4) ≤72 hours from the initial head injury

5) Cooling device or technique available for >48 hours

6) Core temperature ≥36°C (at the time of randomisation)

7) An abnormal CT scan of the brain. This is defined as one that shows haematoma, contusion, swelling, herniation or compressed basal cisterns.

#### Exclusion Criteria

1) Patient already receiving therapeutic hypothermia treatment

2) Administration of barbiturate infusion prior to randomisation

3) Unlikely to survive for the next 24 hours in the opinion of the ICU Consultant or Consultant Neurosurgeon treating the patient

4) Temperature ≤34°C at hospital admission

5) Pregnancy (all female patients of child bearing age who meet the inclusion criteria will undergo a urine pregnancy test. This is performed as part of the screening for eligibility procedure by the investigator or research nurse in the ICU).

### Participant Selection and Enrolment

#### Identifying Participants

Eligible participants are identified by nursing and medical staff on the ICU.

#### Consenting Participants

Eligible patients for this study must have raised ICP despite stage 1 treatment options for the management of head injury. Stage 1 treatment options include sedation and ventilation therefore participants are not be able to give informed consent themselves.

The patient and relative information sheets were designed in consultation with patients who had suffered a TBI and their relatives. Consultations took place at the drop-in centre for the Edinburgh Headway Group, a registered charity for brain injured patients http://www.edinburghheadway.org.uk.

#### Screening for Eligibility

A screening log is completed for all eligible patients. Data including inclusion criteria met, exclusion criteria not met and date consent obtained are collected on this form. It is kept in a locked cabinet at the centre. This data are slso entered in the trial database via the electronic case report form (CRF) in order for the trial office to monitor recruitment and/or refusal rates at each site.

### Randomisation

Patients should be randomised as soon as possible after meeting the inclusion criteria. The randomisation of participants to hypothermia or standard care is undertaken using either a central internet based randomisation service or a telephone randomisation service depending on the available technology at each site.

Treatment allocation is minimised using the following baseline covariates (but includes a random element to ensure allocation concealment):

1) Trial Centre

2) Age < or ≥45 years

3) Post-resuscitation Glasgow Coma Score motor component 1-2 or 3-6

4) Time from injury < or ≥12 hours

5) Pupils: both reacting or 1 or neither reacting

#### Treatment Allocation

It is not possible to blind local investigators to allocation as it is obvious clinically which patients are receiving hypothermia e.g. equipment required, patient temperature, blood results, fluid requirements. Blinding of outcome data assessment is however be ensured as the GOSE questionnaire is posted to participants by the coordinating centre.

#### Premature Withdrawal

Participation in any research trial is voluntary and therefore the participant or their legal representative may wish to withdraw from the trial at any point. If this is the case, it should be made clear on a Premature Withdrawal Form whether any previously collected data may still be used for the analysis and which part of the trial the patient is being withdrawn from:

1)Withdraw entirely - the hypothermia intervention is safely terminated, no further data is collected and previous data collected will not be used in the analysis.

2)Withdraw entirely - no further data is collected and the intervention is safely terminated but data previously collected may be used in the analysis.

3)Withdraw from the intervention but be willing to be followed up.

4)Withdraw from being followed up only.

If the patient wishes to withdraw from the trial or their legal representative wishes to withdraw them, they are free to do so without giving a reason and without the patient's medical care or legal rights being affected [47]. If however the patient is withdrawn from the study by the doctor in charge of their care on medical grounds, the reason for this withdrawal must be clearly documented in the data collection form and a serious adverse event form completed if appropriate.

### Data Collection and Checking

Daily data collection starts on the day of randomisation (baseline) for all patients and continues until the ICP monitor has been removed. Data is collected using an electronic CRF. This includes the Modified Oxford Handicap Scale which is completed at hospital discharge. Paper copies of all CRFs are available to centres with little or no access to the internet. All CRFs must be completed in English and is managed by Lincoln, Paris. Blinded and patient identifiable data are stored separately in secure databases. All patient identifiable data are stored by the coordinating centre.

#### Follow-up Data

The patient's General Practitioner/Family Doctor is sent a letter by post to inform them of the patient's involvement in the Eurotherm3235Trial.

Patient outcome is assessed 6 months after injury using the GOSE questionnaire. As this is the primary endpoint of the study, it is vital that this information is obtained. If the patient is still in hospital 6 months after the injury, the research nurse or investigator may visit the patient on the ward to go through the questionnaire with them if this is appropriate. If however the patient has been discharged from hospital, the questionnaire is sent to their residing address. A member of the trial team will telephone the patient's family doctor to find out their vital status before any questionnaires are sent to the patient.

It is likely that the patient is unable to complete the questionnaire by themselves due to the nature of their injury. Therefore a letter will also be sent to the person who gave consent for the patient inviting them to help the patient to complete the form and remind them of the study. This is sent at the same time as the GOSE questionnaire is sent to the patient. If a response is not received from the patient within 3 weeks, they are sent the shorter GOS questionnaire with a covering letter.

If there is still no response, and the patient has been discharged from hospital, an attempt will be made to contact them directly by telephone. If the patient lacked capacity at hospital discharge and cannot be contacted directly by telephone, the person who consented for the patient to be enrolled in the study is telephoned and asked to complete the shorter GOS questionnaire over the telephone.

If however the patient regained capacity before hospital discharge and has given consent to continue to be involved in the follow-up phase of the study, yet cannot be contacted directly by telephone, no further contact is made. This process is discussed during the consent procedure.

Staff in Edinburgh work closely with local investigators to obtain data that are as complete and accurate as possible. Key data, such as outcome measures, are 100% double entered into the trial database. Extensive range and consistency checks are further enhance the quality of the data.

### Statistics and Data Analysis

#### Sample Size Calculation

The primary endpoint for this trial is outcome at 6 months measured by the GOSE questionnaire. The main evidence on therapeutic hypothermia has been gathered by six meta-analyses published between the years 2000 and 2008. These included varying numbers of clinical trials and examined each trial based on an assessment of the quality of randomisation and blinding procedures. All meta-analyses found a trend to positive effects of hypothermia on neurological outcome, but statistical significance was reached in only two [37,38].

A major reason for the failure of previous Phase III trials in TBI has been that they have typically been powered to detect an absolute decrease in poor outcome of around 10%. This is an unrealistic target in view of the enormous heterogeneity of the TBI population. A more modest 7% absolute decrease remains clinically relevant and is more realistic as a hypothesised treatment effect (corresponding to an odds ratio of 0.75). Two groups of 815 give 80% power at the 5% significance level (2-sided) to detect a decrease in poor outcome from 60% to 53% and two groups of 900 would give 83% power to detect the same difference at the same significance level. We aim to enrol 1800 patients in the main trial to allow for loss to follow up. Importantly, we have been conservative with the power calculation to reduce the chance of conducting another underpowered trial in this field.

#### Proposed Primary Analysis

A detailed Statistical Analysis Plan (SAP) setting out full details of the proposed analyses will be finalised before the trial database is locked for analysis. The primary analysis will follow these principles:

• The analysis will be undertaken on the 'intention-to-treat' principle.

• The estimated treatment effect will be presented along with its corresponding 95% confidence interval.

• The analysis of the primary outcome measure, the 6 month GOSE, will exploit the ordinal nature of the outcome scale. It is currently an active research question in both TBI and stroke trial methodology which approach to use to analyse such ordinal outcome scales, the two main options being 'shift analysis' and the 'sliding dichotomy'. The preferred approach will be declared in the SAP, taking into account the results of current on-going methodological research.

• The primary analysis will be adjusted for key baseline covariates, to be specified in the SAP. The unadjusted analysis will also be presented as a sensitivity analysis.

• All interim efficacy analyses reviewed by the independent Data and Safety Monitoring Committee will be interpreted according to the strict Peto-Haybittle guideline so that no adjustment is required to the final p-value to allow for the multiple testing.

#### Other Planned Analyses

*A priori *sub-group analysis will be presented testing the relationship between minimisation factors including: age <45 years, admission post resuscitation Glasgow Coma Score motor response <2, time from injury <12 hours and outcome. The analysis will test for interaction effects, and stricter levels of statistical significance (p < 0.01) will be sought, reflecting the exploratory nature of these subgroup analyses. Only the primary outcome measure will be used in these analyses.

Other exploratory and observational studies will be conducted by some centres. These sub-studies will be run by local Investigators and will require approval by the trial management and steering committees together with further ethical approval. All sub-studies must also have secured external funding.

#### Economic Analysis

The undertaking of economic data collection and analysis will be dependent on obtaining external funding. Details of this analysis will be added when external funding is obtained.

### Adverse Events

The ***only ***serious adverse events to be collected are:

• Bleeding - defined as a new haemorrhage requiring ≥2 units of packed red cells.

• Cardiovascular instability - defined as a systolic blood pressure <90 mmHg for ≥30 minutes [37]. Terminal hypotension is not be collected.

• Thermal burns >5% of body surface area using the Lund-Browder Chart.

• CPP < 50 mmHg for ≥15 minutes.

### Trial Management and oversight arrangements

#### Project Management Group

The trial will be coordinated by a project management group, consisting of the grant-holder and Chief Investigator in Edinburgh, Trial Managers and advisers.

#### Trial Management

The trial office is associated with the Edinburgh Clinical Trials Unit in the University of Edinburgh and gives day to day support to the clinical centres. Trial office staff are responsible for all aspects of trial management. These responsibilities include providing research advice and support to all centres, ensuring the timely completion of CRFs in collaboration with all centres, data checking and analysis. The trial office staff are also be responsible for the production of progress reports for the data and safety monitoring committee (DSMC), trial steering committee (TSC), ethics committees and the European Society of Intensive Care Medicine (ESICM) who are funding the study. Publication and dissemination of the study results will be coordinated by the Edinburgh Clinical Trials Unit in collaboration with the Chief Investigator and Principle Investigators.

A senior Trial Manager oversees the study and is accountable to the Chief Investigator. Two Trial Managers supervise the day to day conduct of the trial, including: initiation of trial centres, ensuring training records are maintained and updated, supervision and support of all trial staff, site visits to all participating centres, regularly liaising with all trial investigators, monitoring of centres and site closures. The Secretary/Data Clerk is responsible for all administrative responsibilities of the trial, including: manual data entering from paper CRFs, monitoring response to follow up questionnaires, following up missing data queries and non responses to questionnaires with the local investigators.

The statistical and scientific integrity of a major clinical trial is enhanced by incorporating three distinct statistician roles: the Study Statistician who undertakes all statistical tasks including formal analysis and reporting of data, the DSMC and an Independent Statistician. This Statistician is truly independent having no trial involvement except producing unblinded interim reports for the DSMC at specified time periods.

Subject to additional funding being obtained, a health economist is responsible for the development of the data collection forms required for the economic evaluation, the analysis of economic data and the preparation of the economic evaluation component of the final report.

An IT programmer has established a database management system for efficient conduct of the trial including the randomisation, timely despatch of questionnaires, automatic form monitoring, data validation and cleaning.

#### Trial Steering Committee

The TSC oversees the conduct and progress of the trial. Other members of the trial management group may attend as observers at the invitation of the Chair of the steering committee.

#### Data and Safety Monitoring Committee

An independent DSMC has been established to oversee the safety of the trial participants. During the period of recruitment to the trial, interim analyses will be supplied, in strictest confidence, to the DSMC together with any other analyses that the committee may request.

In the light of these analyses, the DSMC will inform the TSC if, in the opinion of the committee, the randomised comparison in the trial has provided either:

a) proof beyond reasonable doubt that for all or some types of patients, the intervention is clearly indicated.(or contraindicated) in terms of a net reduction in morbidity and mortality across groups. (Appropriate criteria for proof beyond reasonable doubt cannot be specified precisely. A difference of at least three standard deviations in the interim analysis of a major endpoint may be needed to justify halting, or modifying, such a study prematurely. If this criterion were to be adopted, it would have the practical advantage that the exact number of interim analyses would be of little importance, and so no fixed schedule is proposed (Peto R et al *Br J Cancer *1976; 34: 584-612).

b) evidence that might reasonably be expected to influence materially the care of people who require ICP management in ICU by clinicians who know the results of this and comparable trials.

c) Futility of enrolment.

The TSC will then decide whether or not to modify recruitment to the trial. Unless this happens, the TSC, project management group, clinical collaborators and trial office staff will remain blinded to the interim results. The conduct of the DSMC is according to the DAMOCLES principles [48].

#### Inspection of Records

Principal investigators and institutions involved in the study will permit trial related monitoring, audits, research ethics committee review and regulatory inspection(s). In the event of an audit, the investigator agrees to allow the sponsor, representatives of the sponsor or regulatory authorities direct access to all study records and source documentation.

#### Study Monitoring

The study will be monitored on behalf of the Co-Sponsors by the Trial Managers. Site staff should be available to facilitate the monitoring visits and must ensure that all required documentation is available for review.

Study initiation visits are carried out at all sites before recruitment commences at that site. Site monitoring is carried out in sites that recruit more than 10 patients throughout the duration of the trial. During these monitoring visits, the Trial Manager(s) carry out Source Data Verification of trial data, verification of informed consent forms and ensure the completeness of the Investigator Site File. Site monitoring is not be carried out routinely for sites recruiting small numbers of patients. Central quality control checks of trial data are however be carried out as described in section 6.0. Where central quality control of data identifies a problem with data collection at any site, or if the Chief Investigator and/or Co-Sponsors have concerns surrounding the quality or validity of the trial data at any site, a site monitoring visit will be conducted.

Serious breaches in the study protocol and/or Good Clinical Practice identified through trial monitoring will be notified immediately to the Co-Sponsors and appropriate corrective action will be taken and documented.

## Discussion

The Eurotherm3235Trial is the result of a Delphi exercise surveying ESICM member's views on research priorities and started in January 2009 with protocol development. This trial is therefore unique, being identified as a priority by members of a scientific society and funded, independent of industry, by the same scientific society.

The burden of bureaucracy that investigators now have to face to conduct clinical trials is larger than ever before. One particular aspect of recent legislation delayed the development of the Eurotherm3235Trial database and management of potential legislative impediment (Commission Nationale De L'informatique Et Des Libertés). This has meant that the data server(s) are now hosted in the University of Edinburgh (UoE). The database went live on April 9, 2010 and prior to this, the trial could not randomise patients outside of Edinburgh.

Detailed contracting is now necessary in all academic clinical trials. The contracts required for the Eurotherm3235Trial include:

• the financial contract between ESICM and the UoE;

• the provision of the trial database between Lincoln, ESICM and the UoE;

• clinical site agreements between the UoE and each recruiting centre (including financial agreement);

• separate contracts for the UK and international sites including Italy, Germany, Russia, Hungary and Greece, required to incorporate the Laws of each participating country. International contracts have been translated to a legal standard then similarly back-translated to ensure consistency whichtakes approximately 6 weeks;

• a service level agreement has between the Chief Investigator and the UoE for the data stored in Edinburgh.

The trial has already achieved many milestones. It is registered with the European registry of trials (http://www.controlled-trials.com ISRCTN34555414). Sponsorship has been agreed: the trial is co-sponsored by the UoE and NHS Lothian in the UK and the UoE outside the UK. Insurance for non-negligent harm has been provided by the UoE for European and Australasian centres but the associated high per-claim-deductable for USA centres has slowed progress in North America. Research ethics committee approval was obtained in Scotland and England in June 2009 and Hungary, Eire, Estonia, Russia, Italy and Germany in May-July 2010, and submissions are well advanced in Greece, Portugal and Belgium. National Institutes for Health Research Portfolio Adoption was achieved following a complex and time consuming process in the UK. This does however make the trial more attractive to UK centres. Trial documentation has been translated into various European languages including Italian, French, German, Dutch, Estonian, Russian, Hungarian, Greek, Flemish and Portuguese.

The current emphasis is on developing the recruitment infra-structure, which is ongoing. This effort required an extremely high level of input; however, the Eurotherm3235Trial is in good shape for the ongoing challenge of the recruitment phase as a result of the hard work of the Trial Coordinating Team, together with Investigators, in establishing the recruitment infrastructure. This work will continue to be of highest priority in order to maximise recruitment.

The trial is open to all ICUs with TBI patients and we encourage clinicians to visit the trial website, register an interest and complete a centre survey. The Trial Coordinating Team are available to help you with regulatory submissions.. The results of the Eurotherm3235Trial will be relevant to all those who look after patients with TBI. The trial can only be successful if clinicians and nurses from ICUs worldwide contribute. We thank the large number of Investigators who are contributing to this trial already.

## Competing interests

The authors declare that they have no competing interests.

## Authors' contributions

Eurotherm3235Trial collaborators conceived the study, participated in its design and drafted the manuscript. All authors have read and approved the final manuscript.
